# Systemic and Brain Pharmacokinetics of Milnacipran in Mice: Comparison of Intraperitoneal and Intravenous Administration

**DOI:** 10.3390/pharmaceutics16010053

**Published:** 2023-12-29

**Authors:** Sounak Bagchi, Ehsan Nozohouri, Yeseul Ahn, Dhavalkumar Patel, Ulrich Bickel, Vardan T. Karamyan

**Affiliations:** 1 Department of Pharmaceutical Sciences, Texas Tech University Health Sciences Center, Amarillo, TX 79106, USA; sounak.bagchi@ttuhsc.edu (S.B.); ehnozoho@ttuhsc.edu (E.N.); yeseul.ahn@ttuhsc.edu (Y.A.);; 2 Department of Foundational Medical Studies, Oakland University, Rochester, MI 48309, USA

**Keywords:** pharmacokinetics, brain, plasma, bioavailability, milnacipran, intraperitoneal, LC-MS

## Abstract

Milnacipran is a dual serotonin and norepinephrine reuptake inhibitor, clinically used for the treatment of major depression or fibromyalgia. Currently, there are no studies reporting the pharmacokinetics (PK) of milnacipran after intraperitoneal (IP) injection, despite this being the primary administration route in numerous experimental studies using the drug. Therefore, the present study was designed to investigate the PK profile of IP-administered milnacipran in mice and compare it to the intravenous (IV) route. First a liquid chromatography–mass spectrometry (LC-MS/MS) method was developed and validated to accurately quantify milnacipran in biological samples. The method was used to quantify milnacipran in blood and brain samples collected at various time-points post-administration. Non-compartmental and PK analyses were employed to determine key PK parameters. The maximum concentration (C_max_) of the drug in plasma was at 5 min after IP administration, whereas in the brain, it was at 60 min for both routes of administration. Curiously, the majority of PK parameters were similar irrespective of the administration route, and the bioavailability was 92.5% after the IP injection. These findings provide insight into milnacipran’s absorption, distribution, and elimination characteristics in mice after IP administration for the first time and should be valuable for future pharmacological studies.

## 1. Introduction

Milnacipran hydrochloride is a dual serotonin and norepinephrine reuptake inhibitor. It exists as a racemic blend, with the chemical designation: (±)-[1R(S), 2S(R)]-2-(aminomethyl)-N, N-diethyl-1-phenylcyclopropanecarboxamide hydrochloride. Milnacipran stands as a nontricyclic entity, showcasing balanced efficacy in hindering the reuptake of both serotonin and noradrenaline, while abstaining from direct interaction with other monoamines and receptors [1]. Approved initially in France in 1996 for managing major depressive disorder (MDD), milnacipran has emerged as a therapy for MDD in over 45 countries worldwide, but not in the United States. The drug has secured authorization for fibromyalgia treatment in the United States, but its utilization for this disorder has not been sanctioned in Europe. The most common side effects of milnacipran are nausea and headache, whereas constipation, hot flashes, sweating, weight loss, dizziness, palpitations, and increased heart rate are among less common adverse effects [2]. Compared to other dual serotonin and norepinephrine reuptake inhibitors, in in vitro experiments milnacipran showed significant potency in impeding norepinephrine reuptake—2–3 times more pronounced than its serotonin-blocking action. For example, uptake inhibition (K_i_ value) of [^3^H]-norepinephrine by the human transporter overexpressed in HEK cells was reported to be 68 nM for milnacipran, whereas that of [^3^H]-serotonin uptake was reported to be 151 nM [3]. This unique characteristic distinguishes milnacipran from other SNRIs that exhibit a greater affinity for serotonin reuptake [4,5], although, in vivo, milnacipran shows comparable affinity for both serotonin and norepinephrine transporters [6]. Notably, levomilnacipran, which is an enantiomer of milnacipran ((1S,2R)-milnacipran) and is deemed to be more active than the racemic mixture, is available in the market as an extended-release capsule formulation and is approved by the FDA for MDD but not fibromyalgia [7,8].

While milnacipran continues to be an active subject of experimental and clinical studies focusing on chronic pain and major depression [9,10,11], it is also being evaluated for other disorders, including cognitive impairment after traumatic brain injury [12], apnea during Rett syndrome [13], attention-deficit/hyperactivity disorder [14] and autism spectrum disorder [15]. Curiously, there is a paucity of published studies reporting systemic and brain pharmacokinetics of milnacipran in rodents. In our literature search, we only found one article describing the pharmacokinetics of milnacipran in rats [16] and two additional articles that studied levomilnacipran [17,18]. Based on this, the current study was designed to study milnacipran’s systemic and brain pharmacokinetics in mice and compare IV and IP routes of administration. The IP over the oral route was selected, because most of the published studies in rodents used this route to administer milnacipran [19,20,21,22], but no published PK study is available for this route. In addition, the IP route is deemed better for the chronic treatment of mice in experimental studies and can be extrapolated to estimate the oral dose [23].

## 2. Materials and Methods

### 2.1. Drugs and Reagents

rac-Milnacipran hydrochloride and rac-milnacipran-d_10_ hydrochloride (IS, internal standard) were purchased from Toronto Research Chemicals (North York, ON, Canada). LC-MS grade acetonitrile, water, and formic acid (99%) were acquired from Fisher Scientific (Fair Lawn, NJ, USA).

### 2.2. Animals

Adult male C57BL/6J mice (8–9 weeks, 23–27 g) were purchased from the Jackson Laboratory, Bar Harbor, ME, USA. Mice were housed in ventilated cages with controlled temperature and humidity, and a 12 h light/dark cycle. Food and water were available ad libitum, and animals were habituated to the experimental room for three days before the start of the study. All procedures were conducted according to a protocol approved by the Texas Tech University Health Sciences Center Institutional Animal Care and Use Committee (protocol 21039, approved on 26 July 2023).

### 2.3. Dosing and Sample Collection

Milnacipran, dissolved in saline for injection, was administered at 30 mg/kg dose via IP (5 mL/kg) or tail vein (115–150 µL) injection (*n* = 4 for each sampling time point). This dose of milnacipran is based on published studies documenting consistent pharmacological effects of the drug in various rodent models [19,20,21,22]. Plasma and brain samples were collected 5, 20, 60, 120, and 180 min after the drug administration. The time-points were selected based on our experience in prior studies [24,25], and initial experiments with milnacipran to cover both early time-points and ≥3 half-lives for the last sampling time-point. Mice were deeply anesthetized with isoflurane and decapitated, followed by blood collection in a microtube containing heparin. Plasma was obtained by centrifugation of the microtube for 10 min at 5000× *g* at 4 °C. The brains were quickly removed and delicately cleansed on filter paper with sterile saline, the surface vasculature was removed using gauze tipped in saline, and the forebrains were quickly frozen using dry ice. Plasma and brain samples were stored at −80 °C until bioanalysis [17,26].

### 2.4. LC-MS/MS Method Development and Validation

To develop a new mass spectrometry method, milnacipran and milnacipran-d10 (internal standard) were introduced to QTRAP 5500 mass spectrometer (SCIEX, Foster City, CA, USA) and the instrument was fine-tuned to identify specific molecular transitions that could be used to identify the analytes accurately and selectively in biological samples. To optimize the sensitivity of the analysis, both negative and positive ionization modes were tested, and the positive ionization mode was selected for subsequent studies. A simple method of protein precipitation was used to extract milnacipran and its internal standard (IS) from plasma and brain samples (see “Section 2.6” in Methods for more details). A series of experiments were conducted to refine the chromatographic conditions, especially the composition and characteristics of the mobile phase, with a goal to achieve improved separation and increased signal levels for the analyte and IS. The developed protocol (see “Section 2.6” in Methods for more details) was evaluated for linearity, accuracy, precision, selectivity, and recovery similar to earlier studies [27]. A calibration curve for milnacipran was constructed by plotting the peak area ratios of the analyte to the IS against analyte concentrations ranging from 7.81 to 1000 ng/mL for plasma and 7.81 to 1000 ng/g for the brain. Linearity was determined using the least-squares method with a 1/x weighted factor, and it was evaluated based on the correlation coefficient (r) [28]. The limits of detection (LOD) and lower limit of quantification (LLOQ) were determined using the calibration standards. The LOD was established at a signal-to-noise ratio of 3, while the LLOQ was defined as the lowest quantifiable concentration with a signal-to-noise ratio of 10, ensuring precision within ±20% and accuracy between 80% and 120% [29]. Precision and accuracy of the method were evaluated in mouse plasma samples spiked with four different concentrations of milnacipran: 7.81 ng/mL (LLOQ, lower limit of quantification), 25 ng/mL (LQC, lower quality control), 400 ng/mL (MQC, middle quality control), and 800 ng/mL (HQC, higher quality control). The samples were analyzed in five replicates on the same day (for intra-day precision and accuracy) and on three independent days (for inter-day precision and accuracy). Concentrations of milnacipran in the samples were determined using daily calibration curves, and precision was expressed as the coefficient of variation (CV%) [28,29]. The aim was to achieve accuracy and precision within ±15% of the nominal concentration, with a CV of ±20% for the LLOQ [27]. In the same way, the intra-day precision and accuracy of the method were evaluated in mouse brain homogenates spiked with three different concentrations of milnacipran: 25 ng/g (LQC, lower quality control), 400 ng/g (MQC, middle quality control), and 800 ng/g (HQC, higher quality control). In addition, recovery (extraction) of milnacipran from plasma and brain samples was determined at three different concentrations: lower (25 ng/mL or ng/g), middle (400 ng/mL or ng/g), and higher (800 ng/mL or ng/g), expressed as the mean area of each QC solution added before sample preparation divided by the mean area of the same QC solution added after sample preparation.

### 2.5. Preparation of Standards and Quality Control (QC) Samples

Primary stock solutions for milnacipran and its internal standard (IS) were prepared in methanol at 1.00 mg/mL concentration. The primary stock solutions were aliquoted into smaller volumes and stored in a −80 °C freezer. Subsequently, working stock solutions for milnacipran and IS were prepared at 10,000 ng/mL concentration in methanol. The spiking solutions of IS were prepared at 20 ng/mL in 100% acetonitrile. Both the working stock solutions and IS spiking solutions were stored in a fridge (2–8 °C) for up to 4 weeks. All stock and working solutions were allowed to equilibrate at room temperature before use. For method development, milnacipran (from the working stock solution) was added to plasma isolated from intact mice to achieve 1.95, 3.90, 7.81, 15.62, 31.2, 62.5, 125, 250, 500, and 1000 ng/mL for analytical standards, and 7.81, 25, 400, and 800 ng/mL for quality control standards. Similarly, homogenates from intact mouse brain in water (1:10, *w*/*v*) were mixed with milnacipran working stock solution to achieve 1.95, 3.9, 7.81, 15.62, 31.2, 62.5, 125, 500, and 1000 ng/g for analytical standards, and 25, 400, and 800 ng/g for quality control standards. 

### 2.6. Sample Processing and Bioanalysis

The brain samples were homogenized at 1:10 (*w*/*v*) in LC-MS-grade water using BioSpec Tissue-Tearor Homogenizer. The plasma sample was diluted 10-fold (*v*/*v*) in LC-MS-grade water. The plasma samples and brain homogenates were subjected to protein precipitation using ice-cold acetonitrile (1:4 ratio) containing 0.1% formic acid along with the IS (10 ng/mL). The samples underwent continuous vortexing for 5 min, followed by centrifugation at 12,800× *g* at 4 °C. The resulting clear supernatant was used for injection into the mass spectrometer. LC-MS/MS analysis was conducted using a QTRAP 5500 mass spectrometer (SCIEX, Foster City, CA, USA) connected with a SHIMADZU Prominence LC system (Kyoto, Japan), consisting of an LC-30AD solvent delivery unit, a DGU-20A3R degassing unit, a CTO-30A column oven, and a SIL-30AC autosampler. Chromatographic separation was achieved on an EVO-C18 100Å column (1.7 µm i.d., 50 × 2.1 mm). The mobile phase comprised 0.1% formic acid in water (A) and 0.1% formic acid in acetonitrile (B) at a flow rate of 0.3 mL/min. The gradient elution was programmed as follows: 0–0.10 min, 10% B; 0.10–1.00 min, 90% B; 1.00–2.00 min, 90% B; 2.00–2.10 min, 10% B; and 2.10–3.00 min, 10% B. The autosampler and column temperatures were 4 °C and 40 °C, respectively. The injection volume was 2 μL. The mass spectrometric detection of the analytes was executed using multiple reaction monitoring (MRM) with an electrospray ionization (ESI) source in positive ion mode. The conditions were curtain gas, 35 psi; ion spray voltage, 5500 V; temperature, 500 °C; ion source gas 1, 50 psi; and ion source gas 2, 55 psi. The precursor ion for milnacipran (MH^+^) was monitored at *m*/*z* 246.8, and a fragment at m/z 100.1 was chosen as the product ion. For milnacipran-d_10_, which served as the IS, the MH^+^ was monitored at *m*/*z* 257.2, whereas a fragment at *m*/*z* 110.2 was monitored as the product ion. The declustering potential (DP) was 46 V for both the analyte and IS, the entrance potential (EP) was 10 V, the collision energy (CE) was 25 V, and the collision cell exit potential (CXP) was 8 V for both. Data acquisition and processing were carried out using Sciex Analyst (version 1.7) and MultiQuant software (version 3.0), respectively.

### 2.7. Statistical and Pharmacokinetic Analyses

The pharmacokinetic parameters were assessed using standard non-compartmental analysis [30]. Microsoft Excel and GraphPad Prism 8.4.3 software were used for calculations and graphical presentation. The apparent first-order terminal rate constant (K_el_) with 95% confidence interval (95% CI) was obtained in Prism by linear regression of log-transformed plasma concentration versus time data. The linear trapezoidal model was used for both IV and IP administration to determine area under the curve from time 0 to 3 h (AUC_0-3_). AUC_0-∞_ (area under the curve from time 0 to infinity) was obtained by extrapolation to infinity, using the mean concentration at the terminal time point and K_el_. The standard errors (SE) of AUC_0-3_ and AUC_0-∞_ were calculated applying Bailer’s method for sparse data [31]. C_max_ and T_max_ values after IP administration are based on actual measurements for the noted sampling time-points. The terminal T_1/2_ with CI was calculated as 0.693/K_el_. The absolute bioavailability of IP-administered milnacipran was calculated as the ratio of the AUC_0-∞_ in plasma after IP vs. IV administration. For the IV route, V_d_ was calculated using the formula Dose/C_0_, and clearance (Cl) was determined as V_d_*K_el_. The MRT was determined using the formula AUMC/AUC_0-∞_ for IP and IV administration routes [32,33,34]. With sparse data sampling, no meaningful standard errors for the latter derived parameters can be calculated. To account for the residual blood in the brain vasculature and the amount of milnacipran in this fraction [35], a simplified correction model, suggested by Fridén and colleagues [35] (Equation (14)), was used to calculate the brain concentration of milnacipran. In these calculations, an experimentally determined value for the mouse brain vascular volume of plasma (9.12 µL/g) was used, as reported in our earlier publication [36]. AUC_0-3_ for brain concentrations was calculated by the linear trapezoidal method.

## 3. Results

### 3.1. LC-MS/MS Method Development and Validation

**Figure 1:**
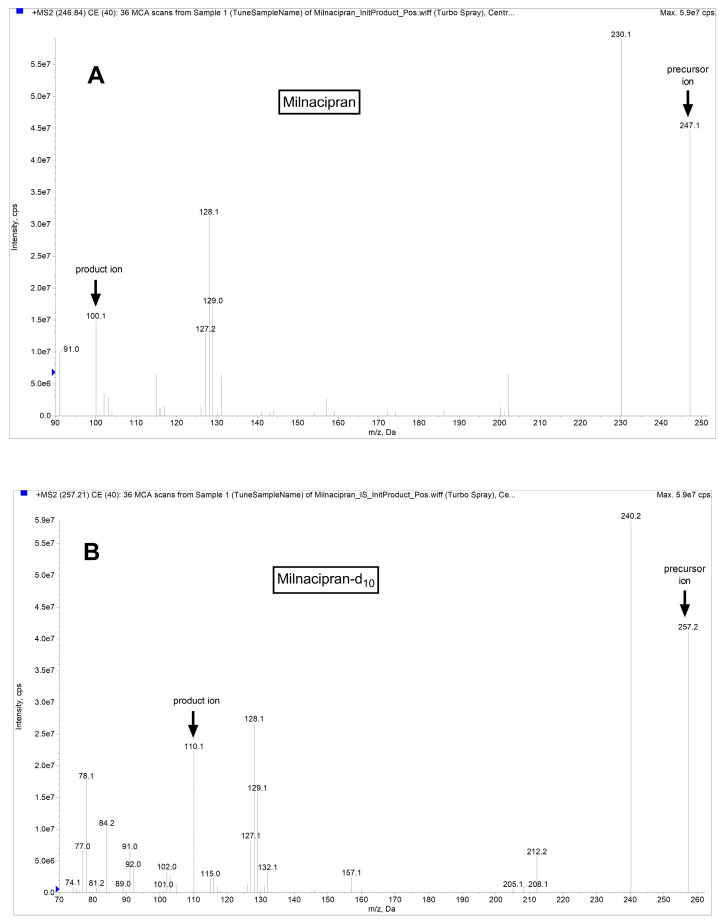
Representative mass spectra for milnacipran (**A**) and IS milnacipran-d_10_ (**B**).

**Figure 2:**
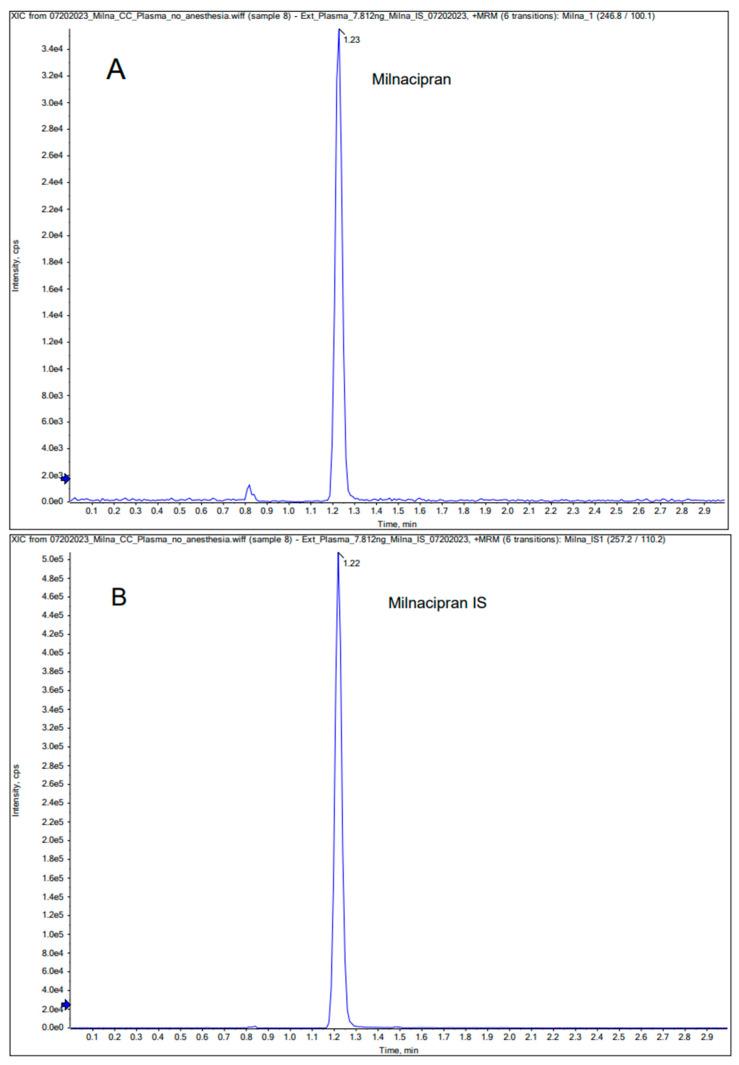
Representative chromatograms for milnacipran (**A**) and IS milnacipran-d_10_ (**B**) in plasma.

In the Q1 full scan mode, we identified the protonated precursor ions [M+H] ^+^ at 246.8 for milnacipran and 257.2 for milnacipran-d_10_. Subsequently, in the MS2 scan mode, we selected ions at *m*/*z* 100.1 (milnacipran) and 110.2 (milnacipran-d_10_) as the product ions. Thus, the selected transitions for quantification were *m*/*z* 246.8/100.1 for milnacipran and *m*/*z* 257.2/110.2 for milnacipran-d_10_. Mass spectra and chromatograms for milnacipran and the IS are shown in Figure 1 and Figure 2. The results of the linearity analysis for a range of milnacipran concentrations are summarized in Table 1. The values for the limit of detection (LOD) and lower limit of quantification (LLOQ) are also provided in Table 1. Precision and accuracy data are summarized in Table 2. Notably, the %CV for intra- and inter-day precision consistently was below 15% across all concentrations of the analyte. Accuracy estimates fell within the range of 95% to 110%, based on comparison of the estimated concentrations to actual values at each QC concentration. The recovery of milnacipran from spiked samples ranged from 92.0% to 108.2% (Table 3) for plasma, respectively, for the above-noted concentrations. The intra-day accuracy results varied from 97.3% to 99.8% for the brain (Table 4). Similarly, for the brain, the recovery percentages were 80.3%, 97.2%, and 92.9%, respectively, for the above-noted concentrations (Table 4).

### 3.2. Pharmacokinetic Evaluation after IP and IV Administration

**Figure 3:**
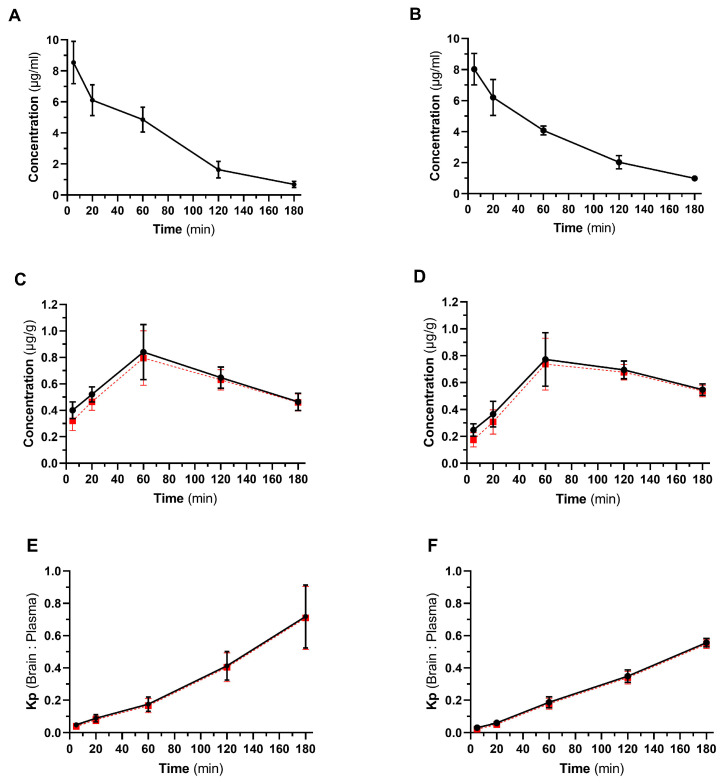
Pharmacokinetic profile of milnacipran after IP and IV administration in mice. Panels (**A**,**B**), time course of milnacipran plasma concentration after IP (**A**) and IV (**B**) administration. Panels (**C**,**D**), time course of milnacipran brain concentration after IP (**C**) and IV (**D**) administration. Panels (**E**,**F**), calculated K_p_ values (brain-to-plasma ratio) for IP (**E**) and IV (**F**) administered milnacipran (30 mg/kg, *n* = 4 for each time point, mean ± SD are presented). Red dash lines represent the calculated brain concentrations (panels (**C**,**D**)) and Kp values (panels (**E**,**F**)) of milnacipran after correction for the drug present in the brain vasculature.

In this study, the concentration of milnacipran was evaluated in plasma and the brain throughout a 3 h observation period after IP and IV administration. The temporal profiles of mean plasma concentrations are illustrated in Figure 3A,B, whereas the corresponding temporal profiles of mean brain concentrations are depicted in Figure 3C,D. The calculated brain-to-plasma ratios (K_p_ value) over time for both routes of administration are presented in Figure 3E,F. To account for the residual blood in the brain vasculature [35], a correction model was applied to calculate the brain concentration of milnacipran without the fraction of the drug in the brain vascular volume of plasma (Figure 3).

Table 5 summarizes the key pharmacokinetic parameters characterizing the plasma pharmacokinetic profile of milnacipran for both administration routes. Irrespective of the route of administration, most calculated parameters were similar. The main exception was the calculated plasma half-life of the drug, which was 42.5 min (95% CI: 36.2 to 54.6 min) for the IP route and 59.2 min (95% CI: 54.4 to 64.1 min) for the IV route. After IP administration, milnacipran’s absolute bioavailability (F%) reached 92.5%. 

Table 6 summarizes the main pharmacokinetic parameters characterizing the brain pharmacokinetic profile of milnacipran for both administration routes.

## 4. Discussion

In the current study, the pharmacokinetic profile of milnacipran was evaluated after IP and IV administration in mice. For this, we first developed and validated a LC-MS/MS protocol to accurately quantify milnacipran in biological samples. Pharmacokinetics of milnacipran was studied at 30 mg/kg dose, because this and higher doses of milnacipran consistently showed pharmacological effects in various rodent models following IP administration, whereas at lower doses of the drug, the outcomes varied widely between different experimental studies [19,20,21,22]. The pharmacokinetic profiles of milnacipran in our study were remarkably similar for IP and IV routes of administration, with very close AUC_0-3_, AUC_0-∞_, V_d,_ Cl, and MRT values, and the absolute bioavailability of the IP route being 92.5%. This is very similar to the original pharmacokinetic study carried out in human volunteers, where an early capsule formulation of milnacipran (PF-C1) was compared to the IV-administered drug, and high absolute bioavailability (85–90%) was documented [37]. In this study, the plasma pharmacokinetic profiles of milnacipran demonstrated striking similarity and eventually converged to an indistinguishable state within 2 h post-administration, regardless of the route of administration.

In humans, milnacipran exhibits rapid and efficient absorption following oral administration, with mild first-pass metabolism and ~90% bioavailability [38,39]. Plasma protein binding is low, around 13%, and demonstrates non-saturable behavior. This facilitates the milnacipran’s swift and widespread distribution throughout the body, with a considerable volume of distribution of 5.3 L/kg. Its terminal elimination half-life spans 6 to 10 h, and steady-state levels are achieved within 36 to 48 h [38,39,40]. Food does not affect the absorption of milnacipran, leading to peak plasma concentrations within 2 to 4 h following oral dosing [2]. Milnacipran’s metabolism primarily involves hepatic pathways (limited involvement of CYP enzymes), with ≤30% undergoing glucuronidation, ≤20% undergoing oxidative metabolism, and ≥50% being eliminated unchanged in the urine. Milnacipran can be used without dose adjustment in patients with mild to moderate renal insufficiency (CrCl ≥ 30 mL/min), but in patients with severe renal impairment (CrCl 5 to 29 mL/min), a 50% dose reduction is advised [41].

In our study, the C_max_ of milnacipran after IP administration was documented at 5 min—the earliest time-point that we used, whereas the terminal half-life after IP administration was somewhat shorter (42.5 min, 95% CI: 36.2 to 54.6 min) compared to that of IV bolus administration (59 min, 95% CI: 54.4 to 64.1 min). Our observations based on the IV administration are fairly similar to a report published by Uchida and colleagues [42], who studied the pharmacokinetics of milnacipran in rats at 20 mg/kg dose and reported a half-life of 2.3 h. In the same study, the half-life of milnacipran after oral, intranasal, and intraduodenal administration (at 20 mg/kg dose) was reported to be shorter—76.2, 67.9, and 47.1 min, respectively. Curiously, another research group reported a substantially longer half-life of 6.7 h for IV-administered milnacipran in rats at 4.5 mg/kg dose [16]. Additional evidence in support of our observations, i.e., shorter half-life of milnacipran in rodents, could be drawn from pharmacokinetic studies of levomilnacipran, because both enantiomers of milnacipran have very similar pharmacokinetic profiles [43]. Bundgaard and colleagues have reported a half-life of 37.7 min for levomilnacipran administered subcutaneously in mice at a 2 mg/kg dose [17]. In contrast, another group studying this drug in rats documented a half-life of 2.3 h after oral administration at 50 mg/kg dose [18]. 

In addition to evaluating the pharmacokinetic profile of IP-administered milnacipran for the first time, our study provides details about the brain pharmacokinetics of this drug in mice. We documented the peak concentration of milnacipran in the brain ~60 min after administration of the drug via both routes, and this was accompanied by increasing K_p_ values for at least 3 h. Our results are analogous to what was observed for levomilnacipran in mice after subcutaneous administration [17], albeit the calculated K_p_ values were higher in this study.

## 5. Conclusions

In summary, our study provides the plasma and brain pharmacokinetic profiles of milnacipran after IP administration in mice for the first time. It compares them to the plasma and brain pharmacokinetic profiles of milnacipran after IV administration concluding that the PK profiles for both administration routes are very similar in the mouse. These findings should help in the design and execution of preclinical studies evaluating the efficacy of milnacipran in various disease states.

## Figures and Tables

**Table 1:** Calibration curve for analysis of milnacipran in the mouse plasma and the brain by LC-MS/MS.

Equation	Linear Range	Correlation Coefficient (R^2^)	LLOQ	LOD
y = 0.01185x − 0.01030 y = 0.01874x − 0.03893	7.81–1000 (ng/mL) 7.81–1000 (ng/g)	0.995 0.996	7.81 (ng/mL) 7.81 (ng/g)	3.9 (ng/mL) 3.9 (ng/g)

**Table 2:** Intra-inter day precision and accuracy of analysis in plasma samples.

Intra-Day (*n* = 5)	Conc. ** (ng/mL)	%Accuracy Mean ± SD	%CV	Inter-Day * (*n* = 15)	Conc. (ng/mL)	%Accuracy Mean ± SD	%CV
	7.81	106.0 ± 4.9	4.64		7.81	104.2 ± 9.4	9.02
	25	106.9 ± 0.7	0.66		25	97.6 ± 7.7	7.88
	400	98.4 ± 2.4	2.42		400	98.9 ± 5.7	5.80
	800	105.5 ± 2.7	2.58		800	100.7 ± 4.2	4.20

* The inter-day precision was estimated by calculating the RSD for the analysis of QC samples in five replicates on three consecutive days. ** Conc., concentration.

**Table 3:** Recovery of milnacipran from plasma (*n* = 5).

Concentration (ng/mL)	Recovery % Mean ± SD
25	92.0 ± 5.0
400	93.3 ± 1.6
800	108.2 ± 1.5

**Table 4:** Intra-day precision and accuracy of analysis, and recovery of milnacipran from the brain samples (*n* = 5).

Concentration (ng/g)	%Accuracy Mean ± SD	%CV	Recovery % Mean ± SD
25	97.3 ± 3.3	3.38	80.3 ± 4.97
400	99.8 ± 2.05	2.05	97.2 ± 2.28
800	98.4 ± 1.97	2.00	92.9 ± 1.3

**Table 5:** Pharmacokinetic parameters of milnacipran in the plasma after IP and IV administration at 30 mg/kg in adult male mice.

Pharmacokinetic Parameters (Plasma)	IP Route	IV Route
C_max_ (µg/mL) *	8.53 ± 0.69	-
T_max_ (min)	5	-
K_term_ (min^−1^) **	0.016 (0.013 to 0.019)	0.012 (0.011 to 0.013)
T_1/2_ (min) **	42.5 (36.2 to 54.6)	59.2 (54.4 to 64.1)
AUC_0-3_ (µg.min/mL) *	616 ± 30	627 ± 22
AUC_0-∞_ (µg.min/mL) *	658 ± 31	711 ± 23
MRT (min)	58.3	57.0
V_d_ (L/kg)	3.37	3.65
Clearance (mL/min/kg)	39.5	42.7
Bioavailability (%)	92.5	-

* mean ± SE are shown; ** mean and 95% confidence intervals are shown.

**Table 6:** Pharmacokinetic parameters of milnacipran in the brain after IP and IV administration at 30 mg/kg in adult male mice.

Pharmacokinetic Parameters (Brain)	IP Route	IP Route with Vascular Correction	IV Route	IV Route with Vascular Correction
C_max_ (µg/g) *	0.84 ± 0.11	0.79 ± 0.1	0.77 ± 0.1	0.73 ± 0.1
T_max_ (min)	60	60	60	60
AUC_0-3_ (µg.min/g) *	113 ± 6	107 ± 6	109 ± 5	104 ± 5

* mean ± SE are shown.

## Data Availability

The data that support the findings of this study are available on request from the corresponding authors.

## Abbreviations

PK, pharmacokinetic; IP, intraperitoneal; IV, intravenous; FDA, Food and Drug Administration; LC-MS, liquid chromatography–mass spectrometry; AUC, area under the curve; MRT, mean residence time; C_max_, maximum concentration; T_max_, time to reach C_max_; Cl, clearance; V_d_, volume of distribution; T_1/2_, half-life; CrCl, creatinine clearance; AUMC, Area Under Moment Curve.
